# Searching for management approaches to reduce HAI transmission (SMART): a study protocol

**DOI:** 10.1186/s13012-017-0610-z

**Published:** 2017-06-28

**Authors:** Ann Scheck McAlearney, Jennifer L. Hefner, Cynthia J. Sieck, Daniel M. Walker, Alison M. Aldrich, Lindsey N. Sova, Alice A. Gaughan, Caitlin M. Slevin, Courtney Hebert, Erinn Hade, Jacalyn Buck, Michele Grove, Timothy R. Huerta

**Affiliations:** 1 Department of Family Medicine, College of Medicine, The Ohio State University, 2231 North High Street, Suite 273, Columbus, OH 43201 USA; 2 Division of Health Services Management and Policy, College of Public Health, The Ohio State University, Columbus, OH 43201 USA; 3 Department of Biomedical Informatics, College of Medicine, The Ohio State University, 310-E Lincoln Tower, 1800 Cannon Drive, Columbus, OH 43201 USA; 4 Division of Infectious Disease, Department of Internal Medicine, College of Medicine, The Ohio State University, Columbus, OH USA; 5 Center for Biostatistics, College of Medicine, The Ohio State University, 320G Lincoln Tower, 1800 Cannon Drive, Columbus, OH 43201 USA; 6 The Ohio State University Wexner Medical Center, 134 Doan Hall, 410 W. 10th Ave, Columbus, OH 43210 USA; 7 Administrator of Health System Nursing Quality, Research, Education and Evidence- Based Practice, The Ohio State University Wexner Medical Center, Office 2021, 600 Ackerman Road, Columbus, OH 43202 USA

**Keywords:** Healthcare-associated infections, Hospital, Toolkit, Management practices, Patient safety, Delphi, Qualitative methods

## Abstract

**Background:**

Healthcare-associated infections (HAIs) impact patients’ lives through prolonged hospitalization, morbidity, and death, resulting in significant costs to both health systems and society. Central line-associated bloodstream infections (CLABSIs) and catheter-associated urinary tract infections (CAUTIs) are two of the most preventable HAIs. As a result, these HAIs have been the focus of significant efforts to identify evidence-based clinical strategies to reduce infection rates. The Comprehensive Unit-based Safety Program (CUSP) provides a formal model for translating CLABSI-reduction evidence into practice. Yet, a national demonstration project found organizations experienced variable levels of success using CUSP to reduce CLABSIs. In addition, in Fiscal year 2019, Medicare will expand use of CLABSI and CAUTI metrics beyond ICUs to the entire hospital for reimbursement purposes. As a result, hospitals need guidance about how to successfully translate HAI-reduction efforts such as CUSP to non-ICU settings (clinical practice), and how to shape context (management practice)—including culture and management strategies—to proactively support clinical teams.

**Methods:**

Using a mixed-methods approach to evaluate the contribution of management factors to successful HAI-reduction efforts, our study aims to: (1) Develop valid and reliable measures of structural management practices associated with the recommended CLABSI Management Strategies for use as a survey (HAI Management Practice Guideline Survey) to support HAI-reduction efforts in both medical/surgical units and ICUs; (2) Develop, validate, and then deploy the HAI Management Practice Guideline Survey, first across Ohio hospitals, then nationwide, to determine the positive predictive value of the measurement instrument as it relates to CLABSI- and CAUTI-prevention; and (3) Integrate findings into a Management Practices Toolkit for HAI reduction that includes an organization-specific data dashboard for monitoring progress and an implementation program for toolkit use, and disseminate that Toolkit nationwide.

**Discussion:**

Providing hospitals with the tools they need to successfully measure management structures that support clinical care provides a powerful approach that can be leveraged to reduce the incidence of HAIs experienced by patients. This study is critical to providing the information necessary to successfully “make health care safer” by providing guidance on how contextual factors within a healthcare setting can improve patient safety across hospitals.

## Background

Healthcare-associated infections (HAIs) contribute significantly to the financial and social cost of hospitalization in the USA. The Centers for Disease Control and Prevention (CDC) estimated that over 700,000 HAIs occurred in 2011, with 1 in 25 hospitalized patients receiving a HAI diagnosis daily [1]. Two infections are associated with a high number of deaths: central line-associated bloodstream infections (CLABSIs) and catheter-associated urinary tract infections (CAUTIs). Further, CLABSIs bear the greatest associated financial cost of all HAIs, estimated between US$960 million and US$18.2 billion every year, while CAUTIs have an estimated cost between US$166 million and US$345 million a year [2]. It is therefore no small issue that 65–70% of CLABSIs and CAUTIs are estimated to be preventable if current evidence-based strategies are successfully used [1].

National efforts have focused on both HAI reduction and prevention. In 2009, the CDC’s National Healthcare Safety Network issued the *National Action Plan to Prevent Health Care-Associated Infections: Road Map to Elimination* [3]. This project saw some progress in decreasing HAIs, but failed to meet national infection reduction goals for 2013: the rate of CAUTIs actually increased 6% between 2012 and 2013 [4]. Currently, Medicare requires CLABSI and CAUTI reporting only for infections in intensive care units (ICUs). However, as more than half of HAIs occur outside ICUs [1], reporting for Medicare is expected to expand to all areas of the hospital. The Agency for Healthcare Research and Quality (AHRQ) is currently working to extend CLABSI- and CAUTI-reduction initiatives beyond ICUs.

### CLABSI-reduction efforts

Evidence has shown that CLABSI rates can be significantly reduced by implementing a “bundle” of five clinical practices: full-barrier precautions; chlorhexidine antiseptic and sterile dressing; optimal vein selection; improved hand hygiene; and prompt removal of unnecessary central line catheters [5–8]. This clinical bundle, combined with dedicated line insertion and maintenance teams, checklists to ensure practice consistency, and practitioner education, has led hospital ICUs to see significant and sustained CLABSI rate reductions [9–12]. Given strong evidence supporting program effectiveness, the Joint Commission and Department of Health and Human Services set the goal of “zero CLABSIs” as a policy tool to mobilize hospital stakeholders, resulting in proliferation of coordinated state and local quality improvement (QI) initiatives and widespread implementation of CLABSI-reduction programs [12–16]. However, while some hospitals have virtually eliminated CLABSIs in ICUs, others struggle to attain and/or sustain near-zero rates [9].

In an attempt to address this variation, the Comprehensive Unit-based Safety Program (CUSP)—a formal model for translating CLABSI-reduction evidence into practice—was developed at Johns Hopkins University and disseminated by AHRQ [6, 10]. CUSP helps hospital units assemble a multidisciplinary team to identify why CLABSIs occur in their unit, and to generate solutions. By 2013, the overall rate of CLABSI infections among hospitals implementing CUSP dropped by 41% [17]. Additionally, 68% of units reported zero CLABSIs for at least one quarter, up from 30% at baseline. While these statistics support program efficacy and the feasibility of achieving “zero,” variability across participating ICUs remains [6, 18], raising questions about how to improve and sustain success.

### CAUTI-reduction efforts

Clinical areas of focus to reduce CAUTIs include appropriate use of short-term catheters, timely removal of urinary catheters, and proper catheter care during placement [19, 20]. Following the successes of CUSP for preventing CLABSIs, AHRQ developed a similar toolkit to prevent CAUTIs in hospitals [21]. However, preliminary data from an appropriateness study [22] with 861 registered hospitals from 37 states [23] showed reductions in CAUTIs, but no significant decrease in catheter use.

### The importance of context in HAI prevention

Both researchers and practitioners have suggested that the context of a QI intervention is integral to its success [24–26]. With CUSP, context can include an organization’s patient safety culture, teamwork structures, and leadership involvement in the initiative. Significant inroads have been made to assess the cultural component of the CUSP program, particularly through the Hospital Survey on Patient Safety Culture (HSOPS). CUSP recognizes the importance of considering the context in which HAI-reduction programs are implemented by noting that “Improvement in Safety Culture” is its third objective [27]. Yet despite CUSP’s focus on patient safety culture, significant variation in HAI-reduction outcomes persists across healthcare systems.

Evaluations of CLABSI-prevention programs including the CUSP final report and a post-hoc analysis of the Michigan Keystone project proposed that organizational factors—leadership and management practices—were potential explanations for variation in levels of success [17, 28]. However, the authors did not try to describe these contextual factors [28]. Similarly, a 2014 systematic review of management practices in HAI reduction found that organizational change, provider education, and audit and feedback were management strategies frequently associated with HAI QI success but echoed the call of an earlier 2007 systematic review [29], with both highlighting the need for a toolkit to bundle management practices and guide implementation.

Current national patient safety improvement programs have the potential to facilitate successful HAI reduction. Specifically, Crew Resource Management (CRM), and a related approach, TeamSTEPPS, are teamwork building programs adapted from the aviation industry that focus on the cultural elements of context, specifically communication and teamwork skills among healthcare providers [30, 31]. A growing body of research links CRM to improved patient safety culture and reduced adverse events, including CLABSIs and other HAIs [30, 32–34]. Recent research conducted by members of our study team revealed strong associations between CRM implementation across a large healthcare system and the communication and teamwork domains of patient safety culture, as measured by the HSOPS, and a much weaker link with leadership and management factors [35]. These findings may be the result of extensive toolkits available that focus on the teamwork and communication goals of CRM, but less information to guide hospitals in implementing supportive management strategies.

In response to these gaps, and given variation evident in the success with CUSP, national-level research conducted by the principal investigator (PI) of this proposal [name blinded for review] sought to open the metaphorical “black box” of management practices to better understand the specific strategies that can influence HAI prevention. Using an exploratory, qualitative approach, eight hospitals from the first wave of AHRQ’s CUSP initiative were classified as higher- vs. lower-performing on the basis of success with CLABSI-reduction efforts. By contrasting higher- and lower-performing hospitals, that study improved our understanding of factors that contribute to variable performance in CLABSI-reduction efforts [36].

Based on this research, the PI and her team proposed a set of broad management strategies and outlined general “best practice” components of successful CLABSI-prevention efforts that appeared to contribute to the context for HAI prevention (see Table 1) [36]. These six management strategies were nearly exclusively present in hospitals classified as higher-performing, and absent or appreciably different in lower-performing hospitals.

**Table 1:** HAI management strategies and example “best practice” components

*Aggressive Goal Setting and Support*
Establish the goal of zero CLABSIs and “walk the talk” Establish a budget to support product adoption, education, and communication efforts
*Strategic Alignment/Communication and Information Sharing*
Include CLABSI rate information as part of organization-level scorecard to be reviewed regularly with executives and board Communicate widely and regularly about CLABSI-prevention goals and progress
*Systematic Education*
Continual re-education about CLABSIs as part of broader patient safety efforts Develop structured education and in-service programs
*Inter-professional Collaboration*
Build and sustain positive physician-nursing relationships Hold inter-disciplinary rounds and safety huddles
*Meaningful Use of Data*
Emphasize importance of data by widely and regularly sharing data on CLABSI rates Prioritize development of automated reporting capabilities to support CLABSI monitoring and compliance with protocols
*Recognition for Success*
Provide rewards and recognition for success with CLABSI reduction efforts and ongoing CLABSI prevention If incentive compensation is used, tie a portion to CLABSI prevention goals

### Searching for management approaches to reduce HAI transmission (SMART): from strategies to guidelines

In this SMART project, our goal is to use the set of six management strategies identified in prior work [36] as a framework to characterize specific structural practices and activities that hospitals put into place to support HAI-reduction efforts generally, and in CLABSI- and CAUTI-prevention. Our SMART study is therefore designed to explore variation across management strategies deployed in HAI-prevention efforts, identify and characterize those structural practices that are present in higher-performing hospitals, and bundle those structural management practices in an implementation program for hospitals using a novel information dissemination platform. As a result, this study will result in the development of an evidence-based toolkit and benchmarking system to support the implementation of effective Management Practice Guidelines (MPGs) that address both CLABSI- and CAUTI-prevention in hospitals.

## Methods

**Fig. 1:**
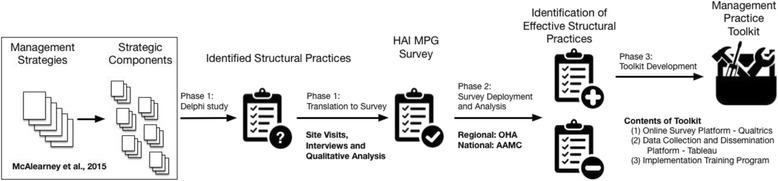
Overview of approach

We propose a 5-year mixed-methods research study, in three phases, to evaluate the contribution of management factors to successful HAI-reduction efforts, specifically in the areas of CLABSIs and CAUTIs. First, a Delphi study followed by targeted site visits and qualitative analysis will enable us to develop measures of the strategic “best practice” components of management strategies as they apply to HAIs. Next, in concert with a psychometrically validated approach to measure development, we will deploy our new HAI MPG Survey across the network of hospitals that are members of collaborating organizations. Analysis of survey results will then guide the construction of a Management Practice Toolkit that includes an implementation plan, an online survey tool, and an organizational dashboard for benchmarking. Figure 1 visually outlines this study approach, which we further explicate below.

### Conceptual framework

This proposal seeks to fill a gap in the literature focused on how systems impact culture in patient safety. The Systems Engineering Initiative for Patient Safety (SEIPS) framework, and its updated SEIPS2 model [37, 38], offers a person-centered sociotechnical system model for the understanding of complex care systems and related healthcare-focused outcomes. The AHRQ-funded SEIPS2 model proposes that elements of the work environment—*tasks*, *people*, *tools*, *organization*—influence work processes that in turn influence patient, professional, and organizational outcomes. Significant work has focused on the study of patient safety culture, or *people,* in the SEIPS2 model. Currently, this concept is measured through the AHRQ HSOPS, a survey of front-line hospital workers addressing workers’ comfort reporting errors, the quality of communication with management about errors, and management commitment to patient safety. Our SMART application proposes to take a step further upstream to determine what components of management practice—*organization* in SEIPS2—can potentially improve patient safety culture, and give hospital leaders and managers actionable steps to improve HAI patient safety culture scores.

### Study hypotheses

Based on our prior work and our conceptual framework, we submit that there exists a diversity of Structural Practices common to high-functioning hospitals, and not present in low-functioning hospitals, that are used in efforts to reduce and prevent CLABSIs and CAUTIs. We believe there will be a pattern of management practices associated with higher-performing hospitals that will provide a differential qualitative experience for the organization. The efficacy of these structural management practices, and potential bundles of practices, can be tested quantitatively across a number of diverse hospitals and leads to our fundamental hypothesis:

*Organizations that exhibit a stronger adoption of certain structural practices (identified and explicated in the course of this study) will have lower HAI rates across clinical practice settings (ICUs and Medical/Surgical Units) within the hospital, where strength of adoption is based on the average implementation of a structural practice across survey respondents within a hospital unit.*

### Study design

Our study focuses on efforts to reduce rates of two HAIs—CLABSIs and CAUTIs. The first two years will involve translating the Management Strategies proposed by McAlearney, et al. [39] into valid and reliable measures of Structural Practices for use as a survey, the HAI MPG Survey, for administration in both medical/surgical units and ICUs. By year 3, we will field the refined HAI MPG Survey nationally across our collaborators. In the study’s final year, we will develop and disseminate a Management Practice Toolkit based on the results of our national level survey data collection; the toolkit will include an Implementation Plan to facilitate use. Below, we provide detail about these study activities, and our Project Timeline presents a quarter-by-quarter depiction of the three study phases.

### Study activities

#### Phase 1: *using a mixed-methods approach, develop valid and reliable measures of Structural Practices associated with the Management Strategies proposed to support HAI-prevention efforts for use as a survey (HAI MPG Survey) in both medical/surgical units and ICUs*

The proposed six Management Strategies outline a series of 21 strategic components recommended for hospitals to implement, but hospitals are currently unable to measure any progress implementing these strategies because metrics associated with these practices have not been operationalized. Our SMART study approaches this problem through the development of robust measurement tools to facilitate improved understanding about the degree to which management practice patterns align to best practice guidelines. The goal of Phase 1 is thus to operationalize the Management Strategies and Strategic Components (see Table 1) proposed to influence HAI-prevention, as detailed in the sections describing the steps of this phase.

A. Explicate the Management Strategies and Strategic Components to Identify Structural Practices (*Participants: approximately 5775 online participants*).
  We propose to use an iterative online engagement strategy based on the Qualtrics [40] platform to explore variation in Management Strategies and Structural Practices focused on HAI-prevention across an initial sample of hospitals. In this step, we will build a survey instrument and deploy a computer-assisted Delphi method to conduct this assessment. The Delphi method is an iterative engagement technique where knowledgeable individuals are systematically asked a series of questions in multiple rounds of queries in five process stages, *Preparation*, *Generation*, *Structure*, *Analysis,* and *Summary*. Throughout this process, the research team will provide summaries of results to participants as findings emerge across the different participants’ perspectives. The Delphi study stages are explained below.
*Stage 1, Preparation* will involve identification of individuals to be included in the Delphi process. The panel of provider and administrative participants will be recruited from our collaborator organizations. We expect to enroll 10% of hospitals across both partner organizations (77 of 765 total facilities) in a purposive sampling approach, seeking to ensure diversity of hospital types and contexts (e.g., critical access, inner city). After hospital enrollment, recruitment emails will be sent out to participating hospitals’ clinical staff. The recruitment email will be from leaders from each member hospital (site contacts), encouraging potential respondents to log into the study site to participate in the Delphi process. Similar online engagement strategies have been used effectively by the National Institutes of Health (NIH), and to create comprehensive conceptual frameworks on a large scale within tobacco and regulatory science [41–43]. To encourage participation in the process, we will offer incentives to respondents through raffles of gift certificates to Amazon.com. Based on response rates from the AHRQ HSOPS process and given the heavily transactional nature of the Delphi data collection methodology and prior experience with this approach, we expect a 30% participation rate leading to approximately 5775 responses across all facilities. Given the narrative nature of this phase, power calculations are inappropriate.
*Stage 2, Generation* uses the proposed six Management Strategies and associated Strategic Components (see Table 1) as the overarching framework for providers, administrators, and patients to respond to prompts designed to explicate these Strategies and their Strategic Components. Participants (recruited in Stage 1) will be directed to respond to question prompts within the Qualtrics software. Posing questions to a variety of participants will enable us to collect a wide range of perspectives on the experience of different management practices that can contribute to HAI prevention. The research team will analyze these submissions and develop narrowed, coherent list of items for use in the next stage.
*Stage 3, Structure* allows participants to sort and rank items from the *Generation* stage. We will use a matrixed approach to question review so that each panel member will receive no more than 10 potential items randomly selected from the bank of potential survey items. The goal of this Delphi study is thus to explicate the range of structural practices used in support of the Management Strategies, and to rank order those practices in terms of two factors: *impact* and *ease of implementation*. While our prior work revealed 21 strategic components related to the six management strategies, it is possible that additional components will be identified through the Delphi process that participants suggest can impact HAI-prevention efforts.
*Stage 4, Analysis* draws upon the sorting and ranking emerging from the *Structure* stage to conduct multi-dimensional scaling and cluster analysis and to create a conceptual map based on the data.
  Finally, *Stage 5, Summarize* uses *Analysis* data to create a summary list of Structural Practices to explore in Step C below.
B. Exploration of Identified Structural Practices (*participants: 26 hospitals; site visits involving 10-15 interviews with key informants*).
  This step will involve qualitative data collection and analysis to inform our understanding of how Structural Practices identified in the Delphi study are operationalized to reduce HAIs in hospitals. This step expands and validates our consensus model from the Delphi process. First, the research team will develop a coherent list of these identified structural practices and incorporate this list into a semi-structured interview guide. This guide will be pilot tested, refined and then used to conduct interviews with nurses, physicians, and administrators across a sample of 26 hospitals. Hospitals will be stratified based on success with CLABSI- and CAUTI-prevention to identify hospitals for these site visits. Using infection rate data from Vizient at the facility level, we will identify 20 hospitals from our collaborators, who are also Vizient members, on the basis of performance in HAI prevention, using a matrix based on CLABSI and CAUTI rates as shown in Table 2.

**Table 2:** Site selection

		CLABSI rates			
CAUTI rates		Lowest	Low	High	Not a factor
	Lowest				3
	Low		5	5	
	High		5	5	
	Not a Factor	3
  Higher-performing hospitals will be defined as having achieved and maintained zero or very low infection rates over the past 12 months. Lower-performing hospitals will be defined as those that had relatively high infection rates in the past year and/or reported increasing infection rates over the past 2 years. We will also select three hospitals with the best performance lowering CLABSI rates over time, and three with the best performance lowering CAUTI rates over time for these site visits. Working through our collaborator organizations, we will approach target hospitals to participate in this study. To ensure geographic variability, we will attempt to recruit “pairs” of higher- and lower-performing hospitals within individual states, as well as consider variability on the basis of different organizational characteristics (i.e., size, number of ICUs, teaching status). While we recognize that not all hospitals approached will agree to participate, past experience with this approach to site recruitment has been very effective [36, 39]; thus, we do not anticipate any problems with hospital recruitment. Two members of our research team trained in qualitative methods will conduct site visits to hold 12–15 in-person interviews with a range of key informants in a variety of roles and responsibilities (e.g., physician, nurse manager, staff nurse, infection preventionist), including individuals working in both ICU and medical/surgical unit settings. All interviews will be audio-recorded and transcribed to permit rigorous qualitative analysis [44] and inform survey development.
C. Survey Measure Development, Validity Testing, and Psychometric Assessment (*participants: 26 additional hospitals, 5 telephonic cognitive interviews with employees of each hospital*).
  In this step, our goal will be to analyze data from the site visit interviews to develop four to five measurement items per management strategy. Using an iterative approach to qualitative data analysis, themes and sub-themes will be identified and characterized [45], helping us to develop items for the survey. This approach identifies specific activities frequently mentioned by interviewees from higher-performing hospitals, and explores the potential absence of these activities at lower-performing hospitals. We will also look at differences in frequently mentioned activities between medical/surgical units and ICUs to determine if we need separate surveys for each of these settings. This iterative approach has been used successfully by members of our research team in prior work [46–48], as well as in other comprehensive studies of best practices [49].
  To test the validity of draft measurement items, we will conduct cognitive interviews in which participants will read each candidate survey item and verbalize their thought process to ensure that their interpretation aligns with the intent of that item. Participants will be recruited from 26 hospitals that were not part of the original sample of hospitals we visited using a similar stratification model. This item-sharing process will involve telephonic cognitive interviews [50, 51] from hospitals recruited via email from our collaborator organizations to key contact persons at the hospitals. From each of the hospitals, 5 targeted individuals will be invited to participate in cognitive interviews based on organizational role (e.g., physician, nurse, administrator, infection control). Participants will comprise a panel for review of draft items.
  We will use a matrixed approach to question review so that each panel member will receive no more than 25 potential items; items will be randomly selected from the bank of potential survey items. Recruitment of multiple participants for cognitive interviews (*n* = 130) will permit each item to be reviewed by multiple panel members. Reviewers will be asked to comment on the items’ understandability, adherence to the targeted concept, and appropriateness for the context to test both face and content validity of the question text. Participants will receive a US$40 gift card as remuneration for their time. We expect that items developed through this process will include both clinical and management content, with the latter including questions linked to CUSP as well as incorporating insights from our prior work [39, 47]. For clinical content, infection control experts on our research team will work with our Research Librarian to identify current measurement tools aimed at assessing clinical aspects of HAI-prevention efforts, including CUSP, with a goal of developing and identifying questions that provide the greatest sensitivity to differences in hospital outcomes.
  Using feedback from the cognitive interviews, we will refine measurement items as needed and develop a draft HAI MPG Survey. This draft survey will then be pilot tested at the research site before more widespread deployment in Phase 2, described next. The process outlined here adheres to the best practices methodologies for instrument and item development outlined by numerous efforts, including NIH PROMIS and the Patient-Centered Outcomes Research Institute (PCORI), where there have been significant efforts to define methodological approaches to developing high-quality, psychometrically-valid instruments.

#### Phase 2*: Administer the HAI MPG Survey within Ohio hospitals to determine the positive predictive value of the measurement instrument as it relates to CLABSI and CAUTI prevention, and expand survey data collection to a national sample of hospitals*

**Fig. 2:**
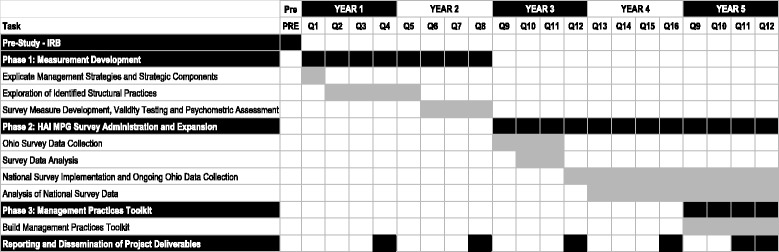
Project timeline
B. Survey Data Analysis
  After survey deployment across Ohio, we will conduct a quantitative analysis of the positive predictive value of the included items to determine which MPG Strategic Components are associated with CLABSI- and CAUTI-reduction success. As part of this process, we will first construct within-hospital unit-level measures of the strength of implementation for each Structural Management Practice, with separate models for CLABSIs and CAUTIs based on unit averages across all responses within each participating unit. We then propose to assess the relationship between unit-level success rates separately for CLABSIs and CAUTIs using a nested logistic regression to control for the fact that units are embedded in hospitals and therefore not independent. In this analysis, each strategic component will serve as an independent variable in logistic regressions - separate for CLABSIs and CAUTIs - with the dependent variable being organizational performance using publicly-reported facility data. Our goal is to assess the positive predictive value for the instruments at both unit and organization levels. We will also construct logistic regressions of multivariate models to determine which practices, and/or bundles of practices, are strongly associated with successful HAI reduction through an assessment of the odds ratios associated with each management practice. Using a nested logistic model, we expect that at the proposed level of engagement we will be able to detect an effect size of less than 0.01 with a power of 0.8 at an alpha level of 0.05; power calculations were conducted using G*Power 3.1.
C. National Survey Implementation and Ongoing Ohio Data Collection
  The research team will expand data collection of the HAI MPG Survey to hospitals nationally (*n* = 10,900 per year, two total years) to ensure that the measures are nationally responsive, as well as continue ongoing survey data collection throughout Ohio hospitals (*n* = 15,400 per year, three total years) with concomitant survey data analysis. National data collection will start at the end of year 3 (see Fig. 2). During this step we will see a significant increase in the number of participating hospitals, and our research team’s efforts will be focused on working with the various hospitals to enable secure, efficient, confidential, and engaged collection of data across the diversity of sites participating. For national survey deployment we will work with our collaborators to engage 10% of member hospitals (*n* = 545), and do not anticipate any issue recruiting this level of participation. Based on the same assumptions above, we expect at least 10,900 survey responses across all sites, each of which would be eligible to participate in the raffle for an Amazon.com gift certificate offered as a participation incentive.
D. Analysis of National Survey Data
  As the final part of Phase 2, we will conduct a quantitative analysis of the longitudinal positive predictive value of the HAI MPG Survey measures, following the approach described above. The national survey is over-powered and is able to detect an effect size of less than 0.01 with a power of 0.8 at an alpha level of 0.05; power calculations were conducted using G*Power 3.1. Our goal is to assess both unit and organizational levels of positive predictive value for the instruments using multivariable models as specified above.

A. Ohio Survey Data Collection
  The HAI MPG Survey will be first deployed across hospitals in Ohio using Qualtrics, an online survey data collection platform. For Ohio, the survey data collection effort will occur in years 3, 4, and 5 (see Fig. 2). Working with the Ohio Hospital Association (OHA), we will secure participation from at least 35% of all Ohio hospitals. OHA represents 220 hospitals and 13 health systems throughout Ohio. Email invitations and reminders will be sent to employees at all levels of participating hospitals. Project champions from each participating hospital will be identified by hospital administration and they will encourage survey participation at their institutions. Given these dynamics, and the success of prior collaborative efforts with OHA, we expect robust participation, and believe that participation from 35% of Ohio hospitals is a conservative assessment, with an anticipated 40% response rate at each site based on AHRQ’s patient safety survey experience. This will result in an average of 200 responses across each site for a total of 15,400 surveys in year 3, with additional survey data collection in subsequent years. Participants at each site will be automatically entered into a raffle for an Amazon.com gift certificate to encourage participation.

#### Phase 3*: integrate these findings into a Management Practices Toolkit for HAI reduction with an organization-specific data dashboard for monitoring progress*

Phase 3 will consist of assembling the Management Practices Toolkit based on the findings from the previous study phases in which we identified and defined metrics to assess evidence-based Structural Management Practices, or bundles of Practices, for addressing CLABSIs and CAUTIs. This toolkit will contain three components: (1) an Online Survey Platform, (2) a Data Collection and Dissemination Platform to permit visualization and dashboarding, and (3) an Implementation Training Program. As detailed in Phase 2, we will implement the survey over years 3–5, collecting data via the Online Survey Platform. Using an online survey tool will facilitate national delivery of the survey at zero cost for interested facilities. Further, instead of sharing Phase 2 results with participating hospitals in a rough format, we propose to develop a dashboard as part of a benchmarking system and provide these resources as part of training guidance that can be encapsulated in a replicable program of implementation and ongoing support. Each of these components is described further below.

1. The Online Survey Platform. Qualtrics is an electronic data collection tool made available nationally. The platform facilitates data availability in Excel, SPSS, and the more generic CSV formats. Qualtrics maintains active development of an Application Programming Interface (API) and will serve as an excellent data collection platform for the HAI MPG Survey.
2. The Tableau Visualization Platform. We propose to develop a Data Collection and Dissemination Platform by linking Tableau Server [52] to Qualtrics via the embedded API to pull data from the system and present benchmarking reports to participating hospitals in real-time. This benchmarking system will allow hospitals to visually compare their performance across all aspects of the SMART tool (i.e., management practice guidelines) against other facilities. Data, gathered in Qualtrics, will be drawn into Tableau and a dashboard system and direct data download capacity will be developed, leveraging existing off-the-shelf tools. The result will be a data collection platform that provides near real-time data for participant hospitals, potentially at the unit level, that is actionable.
3. Implementation Training Program. To facilitate implementation of the toolkit, we propose to package the recommendations along with the supporting survey and benchmarking visualization platform as an integrated program. Encapsulating these HAI-prevention resources in a training program that is part of the toolkit will enable hospitals to operationalize the elements presented in McAlearney et al.’s proposed management strategies [39]. The Implementation Training Program will also include information on the human resources and operational changes necessary to successfully deploy the evidence-based practices.

### Dissemination of the toolkit

In partnership with AHRQ, and similar to other AHRQ patient safety initiatives such as HSOPS and CUSP, we will set up an interactive website to host all three elements of our toolkit. However, unlike the AHRQ HSOPS comparative data dashboard, we will develop an interactive system in which each participating hospital can see their individual data alongside national benchmark data. Beyond Phase 2 data collection, which will utilize the online survey and visualization platform, our intent is to disseminate the link to this online toolkit through our collaborators, and through AHRQ’s extensive dissemination network, as part of Phase 3.

## Discussion

The proposed project is a large-scale study examining the Structural Management Strategies critical to HAI reduction. Current scholarship around HAI reduction has focused on clinical best practices—operationalized as CUSP, among others - and patient safety culture—operationalized as the HSOPS survey. As detailed in our Conceptual Model section, through our use of the SEIPS2 model, the SMART study will address gaps in this area by elucidating management practices that are critical to successful implementation of HAI-prevention programs such as CUSP and/or programs to improve patient safety culture such as CRM/TeamSTEPPS.

Additionally, this project will produce a Management Practices Toolkit. The actionable steps mentioned above will be operationalized as a toolkit that can be implemented alongside the AHRQ CUSP CLABSI and CAUTI toolkits. This new toolkit will expand guidance about general management practices, e.g., beyond identify a unit champion, which comprise the current CUSP toolkits. Specifically, the SMART Management Practices Toolkit will include a plan to guide implementation of evidence-based structural practices within hospitals, and provide information about the associated human resources and financial considerations necessary for practice implementation. The toolkit will also include an online survey, a dashboard to benchmark an organization’s progress, and a set of corrective strategies.

This proposal also employs a particularly innovative Data Collection and Dissemination Platform. By linking Tableau Server to Qualtrics, as we described above, this platform will enable presentation of data and reports to participating hospitals in real-time, allowing organizations to monitor progress in HAI-prevention efforts and be alerted to opportunities for corrective action and organizational change to address identified issues.

### Limitations

We foresee several issues that may create limitations for this study. First, the Delphi process relies on diverse stakeholder participation, leading to the potential for response bias. We believe our sample will provide a group of sufficient size to represent a variety of perspectives, mitigating the risk of response bias. Additionally, we intend to leverage our relationships with collaborating organizations to aid in recruitment, and have included incentives to encourage participation.

Related, as with all survey research, there is the potential for response bias to the survey development in Phase 2. To mitigate this potential, we will validate new measures using a sample drawn from hospitals across the nation, which should further reduce bias. In addition, while the study relies on self-reported data, it is important to note that, similar to AHRQ's approach with HSOPS, practice varies within contexts, and there is no means to assess practice patterns except through the assessment of those engaging in the practice itself.

Also, as described in Phase 2, our initial survey administration and linkage with HAI performance data will take place only in the state of Ohio, which may limit the generalizability of our initial findings. In this initial step, we will collect survey data and link the data to HAI scores, a process that will require agreements with hospitals to provide data in a secure, efficient, and confidential manner that may not be feasible nation-wide. Thus in our initial step, we will leverage the Ohio collaborators to facilitate these agreements and define protocol locally to inform the planned national administration of the HAI MPG Survey.

## Conclusions

The work of this R01 will lead to further efforts to disseminate the HAI Management Practices Toolkit beyond our collaborating partners. We aim to develop a community of practice that shares benchmarking data and feeds survey results back to our study team as well as to other participating hospitals, facilitating ongoing refinements of the tools to support HAI-prevention efforts across hospitals. Building a community of practice will also allow hospitals to learn from each other and spread implementation of the toolkit to promote management practice changes that will improve patient safety.

## Abbreviations

AAMC: Association of American Medical Colleges
AHRQ: Agency for Healthcare Research and Quality
API: Application Programming Interface
CAUTIs: Catheter-associated urinary tract infections
CDC: Centers for Disease Control and Prevention
CLABSIs: Central line-associated bloodstream infections
CMS: Centers for Medicare and Medicaid Services
CRM: Crew Resource Management
CUSP: Comprehensive Unit-based Safety Program
HAIs: Healthcare-associated infections
HSOPS: Hospital Survey on Patient Safety Culture
ICUs: Intensive care units
IDEA4PS: Institute for the Design of Environments Aligned for Patient Safety
MPG: Management Practice Guidelines
NIH: National Institutes of Health
OHA: Ohio Hospital Association
OSU: The Ohio State University
OSUWMC: The Ohio State University Wexner Medical Center
PCORI: Patient-Centered Outcomes Research Institute
PROMIS: Patient-Reported Outcomes Measurement Information System
SEIPS: Systems Engineering Initiative for Patient Safety
SMART: Searching for Management Approaches to Reduce HAI Transmission
